# Diabetic Erythrocytes Test by Correlation Coefficient

**DOI:** 10.2174/1874431100802010105

**Published:** 2008-06-05

**Authors:** A.M Korol, P Foresto, M Darrigo, O.A Rosso

**Affiliations:** 1 Departamento de Matemática y Estadística, Universidad Nacional de Rosario, Facultad de Ciencias Bioquímicas, Suipacha 531, (2000) Rosario, Argentina; 2 Centre for Bioinformatics, Biomarker Discovery and Information-Based Medicine, School of Electrical Engineering and Computer Science, The University of Newcastle, University Drive, Callaghan, NSW, 2308, Australia; 3 Instituto de Calculo, Facultad de Ciencias Exactas y Naturales, Universidad de Buenos Aires, Ciudad Universitaria (1428), Ciudad de Buenos Aires, Argentina

## Abstract

Even when a healthy individual is studied, his/her erythrocytes in capillaries continually change their shape in a synchronized erratic fashion. In this work, the problem of characterizing the cell behavior is studied from the perspective of bounded correlated random walk, based on the assumption that diffractometric data involves both deterministic and stochastic components. The photometric readings are obtained by ektacytometry over several millions of shear elongated cells, using a home-made device called Erythrodeformeter. We have only a scalar signal and no governing equations; therefore the complete behavior has to be reconstructed in an artificial phase space. To analyze dynamics we used the technique of time delay coordinates suggested by Takens, May algorithm, and Fourier transform. The results suggest that on random-walk approach the samples from healthy controls exhibit significant differences from those from diabetic patients and these could allow us to claim that we have linked mathematical nonlinear tools with clinical aspects of diabetic erythrocytes’ rheological properties.

## INTRODUCTION

It is important to appreciate the role of mathematics in the analysis of physiological systems. From the ability to predict the future course of the system behaviour, the related goal of control follows naturally [1]. Probably the greatest appeal of chaos for physiology is the simple observation that so much physiological activity is highly variable, appearing random or noisy. A chaotic system can appear this way as well, but there is an underlying deterministic structure.

Erythrocyte deformability improves blood flow in microvessels and in large arteries at high shear rate. Physiologically, the erythrocyte deformability depends on the surface-volume ratio, internal viscosity and dynamics properties of the erythrocyte membrane.

Ektacytometry is a well established method in which cells, usually erythrocytes, are exposed to increasing shear stress and laser diffraction pattern through the suspension is recorded. The diffraction pattern, which is circular when the mammalian erythrocytes membrane is at rest, becomes elliptical when the cell undergoes shear stress. When laser is applied during creep and recovery process, light intensity dynamically changes along the major axes of the elliptical diffraction pattern. These experimental determinations are carried out with a home made device called Erythrodeformeter [2], which was developed and constructed for rheological measurements of red blood cells subjected to definite shear stress. This fluid shear stress is similar to the one in the capillaries. The corresponding time series (diffracted intensity measured in the major axis of the elliptical pattern under creep or recovery process) can be used in order to obtain same insight of the corresponding associated dynamics under healthy or illness conditions.

In the characterization of erythrocyte viscoelastic properties (time series) corresponding to healthy donors and hematological disorders, nonlinear dynamics tools and correlated random walk approach have been applied [3-7]. Diffractometric data belonging to healthy donors behave as white noise, while data series from different disease were found to be chaotic. Also, evidence of ordinary Brownian motion was found in the case of healthy donors. On the other hand, for samples corresponding to patients with hereditary spherocytosis, dyslipidemic and beta thalassemic a fractional Brownian motion was found [3-7].

In order to compress information contained in the diffractometric data, in such a way that emphasizes the most significant characteristics, we must not merely use observer's judgement but objective methods of analysis.

The clinical interpretation of erythrocytes deformation through the photometrically recorded series obtained measuring the diffraction pattern, attempts to link pathological features with the visual microscopy inspection of the cells samples.

The diabetes mellitus is studied in the present work. Diabetes mellitus induces several changes in the erythrocyte membrane and its cytoplasm, leading to alteration in the deformability. A decreasing trend of deformability in diabetes patients has been reported [8, 9]. Many studies have shown that diabetes mellitus is associated with increased whole blood viscosity and decreased erythrocytes deformability. It has been suggested that these abnormalities in blood rheology may play a causative role in the pathogenesis of diabetic vascular complications [10, 11].

Tools for nonlinear biosignal analysis are quite different from those used in the linear approach. The basic strategy is to determine appropriate characteristics of the recorded signal, which have changed associated with the creep and recovery process of the erythrocytes as the signal unfolds in time. During this process the question about order or chaos arises. To exclude amplitude information, the photometrically time series were normalized to have a variance of unity. All the series undergo Fourier analysis and appropriate smoothing was performed according to a frequency and amplitude dependent algorithms [12]. We also evaluate an essential aspect of the phase space trajectory, which is the Correlation Coefficient proposed by May and co-workers [13], which is a measure of the sensitivity of the process to initial conditions.

The results of the present study suggest that on random-walk approach the samples from healthy controls exhibit significant differences (ordinary Brownian motion) from those from diabetic patients (fractional Brownian motion) and these could allow us to claim that we have linked mathematical nonlinear tools with clinical aspects of diabetic erythrocytes’ rheological properties.

## MATERIALS AND METHODS

### Materials

Blood samples were obtained from ten normal healthy controls and ten diabetic patients. Blood was collected into sterile syringes by using very large bore needles and anticoagulated with heparin. The healthy controls were adults (non-smokers and non-alcoholic individuals), they had no known pathology and were not being medicated. The erythrocyte suspension was prepared by resuspending 50 mm3 of anticoagulated packed cells, resuspended in 4 ml of an isotonic medium of high viscosity. The suspending medium was prepared by diluting 3.1 % (w/v) of Polyvynilpyrrolidone (PVP360-Sigma, MW 360.000) in phosphate buffered saline solution (PBS) (pH 7.4± 0.05, 295±8 mOsm/kg). The viscosity of this medium was adjusted to 22±0.5 mPa at 23°C as measured in a Wells-Brookfield DV-III cone plate viscometer. Collection and processing of the blood samples, for this work, were performed in accordance with the recommendations, of the ICSH Guidelines [14].

### Experimental Setup

A schematic of the Erythrodeformeter [2] is presented in Fig. (**1**). It tests the ability of red blood cells to change their circular shape into an ellipsoidal one, when they are subjected to a well controlled fluid shear stress. This home made device has two plane disks, both of them were made by flint glass, superposed, coaxials, parallels and horizontals. The driving motor is coupled to the lower disk axis by two helicoidal gears that provide a great rigidity to the transmission system, allowing it (rotational disk), to start or stop in rotation, in such a short time (< 1 ms), that it can be considered as instantaneous. The light source is a 5-mW He-Ne laser. When the layer of diffracting cells is perpendicularly transverses by the laser beam, the diffraction pattern can be observed on a ground glass screen. Diffracted intensity corresponding to each principal diameter of the elliptical diffraction pattern falls onto a masked photomultiplier tube (PMT), after passing through a thin straight slot in the mask placed exactly on the corresponding axis of the elliptical pattern.

All units, except the oscilloscope and the PC, are home made equipment. The PMT signal output is either relayed to the micro ammeter or to the oscilloscope. When the PMT is connected to the micro ammeter, two values of electrical current could be measured. They correspond to the long and short axis of the elliptical pattern. On the other hand, the PMT is connected to the oscilloscope through an 8 bit A/D (A/D-C) converter having a memory divided into 32 sectors with 256 bytes each. The conversion delay is 0.1 ms and the converter board has two important controls which must be adjusted for optimal performance; one selects the memory sector to be filled during the data acquisition, the other selects the lapse of time during which a set of 256 data points will be acquired and stored. The whole time series was obtained by concatenation of 20 consecutive files (256 data each) recorded by the Erythrodeformeter under creep and recovery condition. In both processes the stationary state is reached after 147 ms and as the data acquisition is 147 ms = 50 data, we have to delete at least the first 50 data points of the first recorded time series to reach the stationary state. In our work we deleted the first 56 data points. Finally, the time series (M = 5064 data) was normalized (zero mean and variance of unity). In Fig. (**2**) the first files, under creep and recovery conditions, for healthy controls obtained by the Erythrodeformeter are shown.

### Data Acqusition

The erythrocyte suspension is placed between the two flint glass disks, and the driving motor allows the lower disk rotation. Normal erythrocytes constitute a single population having discoidal shape with almost the same size. Light diffraction under Fraunhofer theory conditions may be applied to obtain quantitative information of diffracting particles such as suspended erythrocytes. The cells in dilute suspension, under shear stress take a three axial ellipsoidal shape having the major axis oriented towards the shear field direction. A laser beam traversing perpendicularly a thin layer of the erythrocytes suspension is diffracted producing a Fraunhofer diffraction pattern that is either circular when the cells are at rest or elliptical when they become deformed by a shear stress field.

The start of data acquisition is externally triggered by the eythrodeformeter motor switch. Once the driven motor is switched on, the PMT signal output is read, converted and stored in a pre selected memory sector. Each time the A/D-C completes a conversion and, as soon as, the creep series has been stored, it returns to the initial position ready to start a new cycle of conversion and storage of another set of data into a new memory sector.

A similar process is carried out when the motor is switched off to store a recovery curve (measured while the cells recover their circular shape from the ellipsoidal one). The A/D-C can receive data from the memory and plot them repeatedly on the oscilloscope screen. A 25 pin connector allows the transfer of data from the A/D-C to the PC by an interfaced bus. Data can be stored in a PC disk for numerically processing off line.

## DATA ANALYSIS

### Basic Data Analysis

To exclude amplitude information, all the time series were normalized to have a variance of unity. The mean was set to zero. The photometric series of healthy controls and of diabetic patients underwent Fourier analysis and appropriate smoothing was performed according to frequency and amplitude-dependent algorithms. Having completed the analysis not only on the whole population of experimentally photometrically recorded time series data but also on a pseudorandom one, it was found that all the peaks were congruent. Therefore this method does not distinguish between an aperiodic, a chaotic, or a random signal, and this limits the application of the Fourier transform, leading to other methods.

### The Percentage of False Nearest Neighbour

A very convenient way to reconstruct the dynamics of the process is to unfold the time series by successively higher shifts defined as integer multiples of a fixed lag τ, (τ = *m*.Δt, where *m* is an integer), and taking *N* equidistant points for creep and recovery process, we are able to define the phase space of all the possible states of the system variables under study. We used the technique of time delay coordinates suggested by Takens and co-workers [15]. Each state of the system can be completely represented by a point in this space. A particular set of points can be connected in time leading to an orbit or trajectory that represents the evolution of the system. The set of orbits starting from all possible initial conditions generates a flow in the state space and can be used as a way to visualise the system attractor. However, limitations of such representation of the system include the conditions that every trajectory must be non-intersecting and that different trajectories originating from different initial conditions must not overlap or occupy the same space. This arises from the fact that a point in phase space representing the state of the system is considered to encode all the information about the system, including both its past and future history, which in a deterministic system must be unique. Then, the points of an orbit acquire neighbours in this phase space. These neighbours, among other things, provide the information on how phase space neighbourhoods evolve in time. In an embedding dimension, that is too small to unfold the attractor; not all the points that are close one to another will be neighbours due to the dynamics. Some will actually be far from each other, and simply appear as neighbours because the geometric structure of the attractor has been projected onto a smaller space. Abarbanel and co-workers [16] examined this question with increasing dimensions, until no false neighbours remained. They developed a method for geometrical considerations alone, known as false nearest neighbours (FNN), to find a value for the minimum embedding dimension to correctly analyze the process dynamics. In practice, working in any dimension larger than the minimum required by the data contamination by round off would arise, if the dimension is less than the minimum required there will appear crosses between the trajectories. This could be checked for increasing embedding dimensions for which noise signal ratios, i.e., that is % FNN is less than 5 %. The method of FNN, could be used as a test on measurements from dynamical system that have been corrupted with noise, when the contamination level is low, the residual percentage of FNN gives an indicator of the noise level.

### Random Walk Approach: Correlation Coefficient

In a classical random walk or more generally, ordinary Brownian motion (oBm), past increments in displacement are uncorrelated with future increments, that is, the system has no memory. In such cases, the mean square displacement of a random walker is linearly related to the time interval Δt by the expression:

< Δ_j_ >^2^ = 2. K_j _.Δt,

where: < Δ_j_ > is the mean square displacement and K_j _is a constant.

The constant K is an average measure of the stochastic activity of a random walker. In a correlated random walk, or more generally, fractional Brownian motion (fBm), past increments in displacement are correlated with future increments, hence the system has memory. In such cases, the mean square displacement is generalized by the following scaling law:

< Δ _j_ >^2^ @ Δt^2Hj^,

where: < Δ_j_ >^2^ is the mean square displacement H_j_ = H_j_(s) is the scaling exponent for creep and recovery, and 0 < H_j _< 1.

This scaling exponent quantifies the correlation between the step increments making up the trajectory of a random walker. This is best illustrated by considering the correlation coefficient for Brownian motion, which is given by the expression:

C(s) = 2 (2^2Hj-1^ - 1),

where:

$\begin{array}{c} H_{j}=1/2\Rightarrow C\left(s\right)=0\left(\text{oBm}\right),\\ 0<H_{j}<1/2 or 1/2<H_{j}<1\Rightarrow C\left(s\right)\neq 0\left(\text{fBm}\right). \end{array}$

In order to obtain the correlation coefficient C(s) between the photometric time series of the recovery process, Y(t) and the theoretical Y*(t), we applied May and Sugihara algorithm. We correlated Y*(t), obtained from the series corresponding to the creep process, X(t), with Y(t) which is the recovery one, for the different steps increments s. For details about the method see Ref. [4, 5].

## RESULTS AND DISCUSSION

This is an active work, trying to use time series analysis for practical applications, this includes: identification of those erythrocytes of patients with severe anomalies, early diagnosis and also classification of the disease from the point of view of nonlinear dynamics. The mechanisms through which diabetic disease could induce vascular damage are both metabolic and mechanical. Hemorheological alterations in diabetes are result of changes affecting both red cell intrinsic structure and their interactions with the plasmatic components. Several hemorheological variables could influence and produce an impaired erythrocyte deformability determining an increased flow resistance in the microcirculation. The lipid-protein interaction can change the activity of membrane protein [17]. It was also reported that there were some changes in lipid component, stiffness and fluidity of diabetic erythrocytes membrane [18].

Once we can predict the future, it is natural to try to control that future, in order to guide the system to a preferred state or keep it away from undesired states. These basic goals of understanding, prediction, and control are closely related to the practical clinical goals of diagnosis and treatment, which underlie much of the rationale for research into physiological systems. Understanding a system’s behaviour and how it is altered under pathological conditions is of course a form of diagnosis, and control is effectively just another word for treatment [19]. Random behaviour is as one might expect unpredictable. Thus the question of randomness in a data series is a relative one, and more a question of mixtures of determinism and randomness. Since noise is present in all physical measurements, determining if randomness is inherent in the system dynamics or in the measurement process is not always straightforward.

The simplest and typical first test is the Fourier transform, in Fig. (**3**) two typical results are shown for healthy controls and for diabetic patients: 1) The first one is about the nature of the power spectrum, where the frequency constituents of the signals are the same for both populations, which could represent a 1/f dynamics. 2) The second result is about the intensity, it is bigger on healthy controls than on diabetic patients especially on low frequencies. It could be because healthy control erythrocytes are completely adapted at flow, while on diabetic erythrocytes the lipid protein interaction can change the activity of the membrane protein [18], which was also reported by Muzulu and co-workers [20]. But from the point of view of Fourier analysis it doesn't distinguish between chaos involving a small number of degrees of freedom and white noise, so this limits its application and leads us to turn to another method, notably that of studying phase space trajectories, which offers significant advantages.

Certainly, the use of a nonlinear quantifier is not intended to replace conventional analyses, but to provide further insights into the underlying erythrocytes deformation mechanisms. In order to reconstruct the process dynamics we define the recovery and creep phase space using the technique of delay coordinates suggested by Takens and co workers. The phase space dimension was chosen applying Abarbanel method of false nearest neighbours. Thus, we obtained for four different and increasing embedding dimensions the %FNN and the results were: for *m *= 3 then %FNN = 19.74; for *m* = 4 then %FNN = 14.19; *m *= 5 then %FNN = 9.08; *m* = 6 then %FNN = 6.17, so we generated two phase space on delay coordinates for creep and recovery and one for the theoretical recovery space obtained from the creep one, all of them with *m* = 7.

**Fig. (4):**
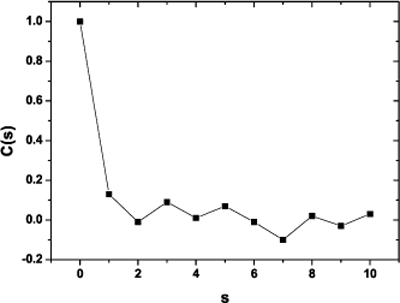
Correlation Coefficient for a pseudoaleatory time series. The length of the time series is equal to the photometric time series analyzed.

Applying May algorithm to these phase space for the first nine steps s of the process we found the Correlation Coefficient C(s). Before computing any correlation coefficient for diffractometric data one should carefully inspect the data and use conventional techniques to extract as much and correct information as possible. So, in order to test the method and the proposed algorithm we contrasted it with a time series of pseudo random numbers (Fig. **4**) as long as the one which corresponds to the photometric time series and it was found to be white noise. This is a random process in which the values at each time are statistical independent from each other. There is a perfect correlation of the signal with itself; and there is no correlation at the entire signal with any shifted version. The process has no memory; past values have no impact in subsequent values as it is expected on a pseudo random time series.

**Fig. (5):**
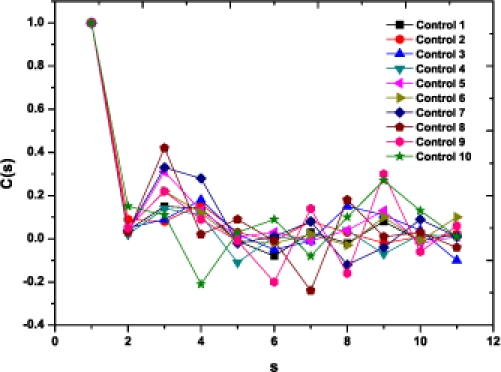
Correlation Coefficient corresponding to the ten healthy control samples.

**Fig. (6):**
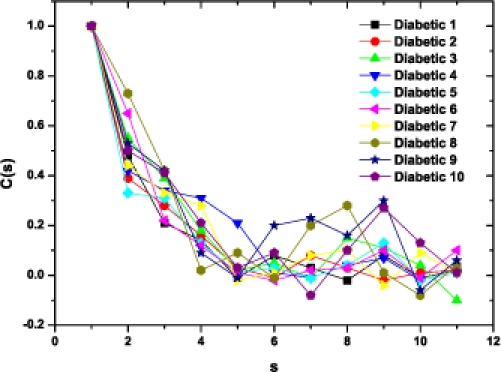
Correlation Coefficient corresponding to the ten diabetic samples.

As a preliminary indication of the possible clinical utility of this technique we have included (Fig. **5**) the linear-linear plots of the correlation coefficient when the diffractometric data belongs to healthy controls. This method was also applied to diabetic erythrocyte samples (Fig. **6**). In Figs. (**7**) and (**8**) the corresponding grand averages are given.

The results were very different comparing healthy controls and diabetic samples. On healthy controls, it could be ordinary Brownian motion (oBm), where statistical properties such as invariance or range are not related at all. On the other hand on diabetic patients, it would be fractional Brownian motion (fBm), it is fractal in the sense that self-similar, in an statistical sense, that is statistical properties are related over different time scales by a way of a power law, in other words, the stress process gives us some special information of the relaxation one in a short time and the series exhibit a great sensitivity to initial conditions.

There are many descriptions of the typical activity that accompanies the deformation but few detailed or quantitative analysis. On the other hand, the quantification of the recorded signals gives the possibility of studying the interactions among different anatomical structures, and the viscoelastic properties of several million of shear elongated cells.

One important finding relates on the complexity of the erythrocytes dynamics, using complexity in a rather loose manner, meaning more noise-like instead less regular, is to get insight in the process. This study found that complexity decreased with disease. In other words, there is such thing as healthy variability. A decrease in this variability can indicate a decrease in health, and variability can endow a system with flexibility and hence the ability to respond and adapt to environmental stressors [21].

Whether this variability is random or chaotic was a key in this study. One of the hopes of the recent application of nonlinear dynamical methods to physiology is that they could provide a general mathematical framework that has been missing from this traditional rather qualitative field.

In this respect, we think that introducing quantifiers derived from nonlinear dynamics could help with the description of the different red cell networks that could put at stake the cytoplasm and membrane interactions.

Moreover, the present results open the possibility of applied novel techniques, like resent methodology based in Information Theory, the causality plane Entropy - Statistical Complexity for a quantitative characterization of these behaviors [22]. Works in this direction, and also further studies with larger and well defined patient populations are in process in order to attain a better validation of the method.

## Figures and Tables

**Fig. (1):**
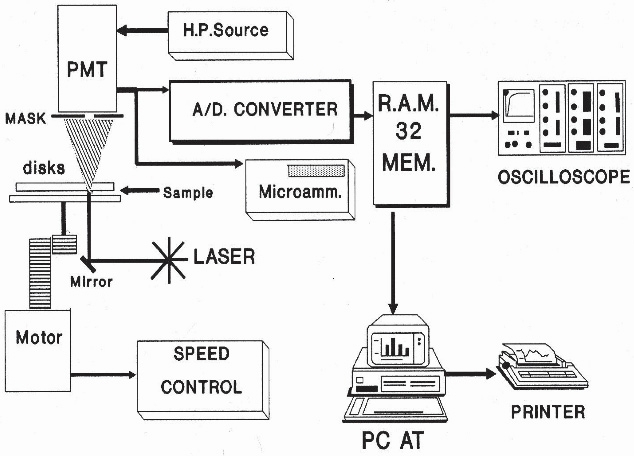
Erythrodeformeter.

**Fig. (2):**
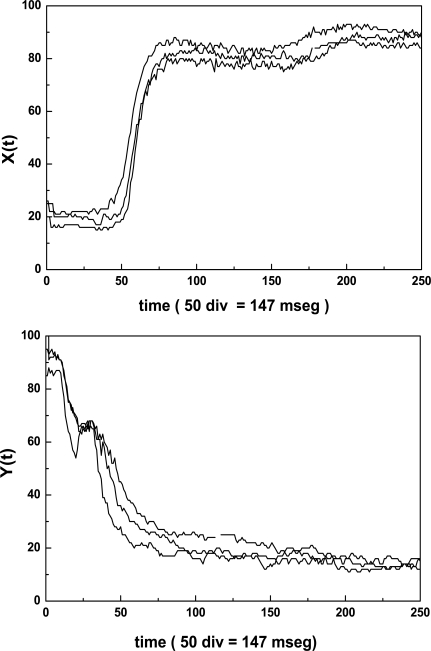
Typical creep X(t) and recovery Y(t) curves of diffracted intensity measured along the major axis of the elliptical pattern corresponding to first file recorded by the erythrodeformeter. Note that the first 56 data points represent the transitory regime.

**Fig. (3):**
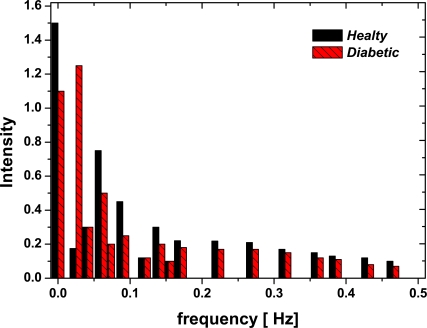
FFT – Fourier Power Spectrum (Intensity *vs* Frequency) for one Diabetic patient and one Healthy Control samples.

**Fig. (7):**
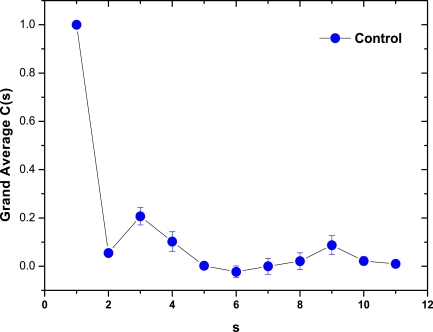
Correlation Coefficient Grand Average for healthy control samples (mean ± SE).

**Fig. (8):**
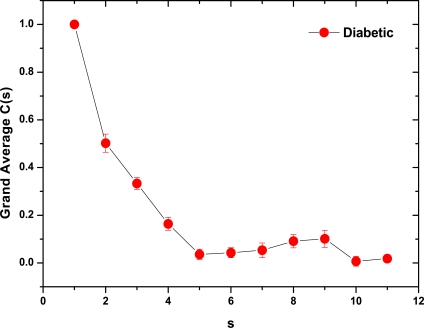
Correlation Coefficient Grand Average for diabetic samples (mean ± SE).
